# Progress in Immunization Information Systems — United States, 2012

**Published:** 2013-12-13

**Authors:** Cristina Cardemil, Terence Ng, Laura Pabst

**Affiliations:** Immunization Svcs Div, National Center for Immunization and Respiratory Diseases, CDC

Immunization information systems (IIS) are confidential, computerized, population-based systems that collect and consolidate vaccination data from vaccination providers that can be used in designing and sustaining effective immunization strategies (1,2). To monitor progress toward achieving IIS program goals, CDC annually surveys immunization program grantees using the IIS Annual Report (IISAR). Results from the 2012 IISAR, completed by 54 of 56 grantees, indicate that 86% (19.5 million) of U.S. children aged <6 years, and 25% (57.8 million) of U.S. adults participated in IIS. Eight of 12 minimum functional standards for IIS published by the National Vaccine Advisory Committee (NVAC) (3,4) have been met by ≥90% of grantees. During 2011–2012, progress was also made in meeting three additional functional standards, including the presence of core data element fields, timeliness of vaccine records, and Health Level 7 (HL7) messaging, and will be monitored in new functional standards for IIS published in 2013 (5). Several new and ongoing initiatives, including interoperability between IIS and electronic health records (i.e., ensuring systems can work together and exchange information), the use of IIS to support vaccine ordering and inventory management, the use of two-dimensional barcodes to record vaccination information (1), and collaboration with pharmacies, federal agencies, and other adult vaccination providers, will support further progress in meeting functional standards and enhance reporting of adult vaccinations to IIS.

Of the 56 immunization program grantees (50 states, five cities,* and the District of Columbia [DC]), 2012 IISAR data† were available for 54 grantees. DC did not report and New Hampshire was not eligible because it did not have an IIS in 2012. The self-administered survey asked about participation in IIS, data quality indicators, and IIS functionality (e.g., interoperability with electronic health records).

## Child and Adult Participation in IIS

Child participation was defined as having two or more vaccinations for children aged <6 years documented in an IIS. Adult participation was defined as having one or more vaccinations administered to adults aged ≥19 years documented in an IIS. Participation was calculated by dividing the number of children or adults in an IIS who met their age group and vaccination criteria by the 2012 U.S. Census estimate of the same age group in the grantee’s geographic area (6). National estimates were calculated by summing the number of children or adults reported to be participating and dividing by the U.S. Census estimate for the total population for that age group.

**FIGURE 1:**
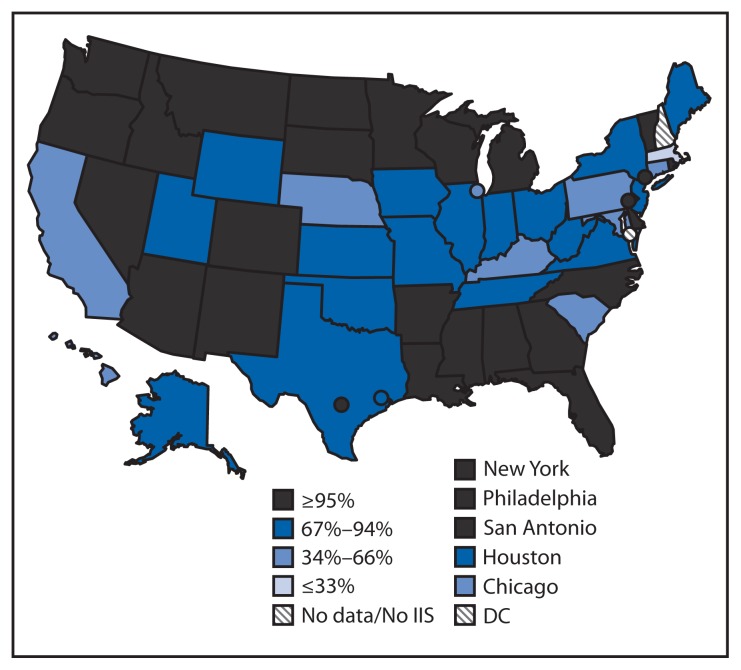
Percentage of children aged <6 years participating in an immunization information system (IIS)^*^ — United States, five cities,^†^ and the District of Columbia (DC), 2012 ^*^ Child participation is defined as having two or more vaccinations for children aged <6 years documented in the IIS. National child participation = 86%. ^†^Chicago, Illinois; New York, New York; Philadelphia, Pennsylvania; Houston, Texas; and San Antonio, Texas.

**FIGURE 2:**
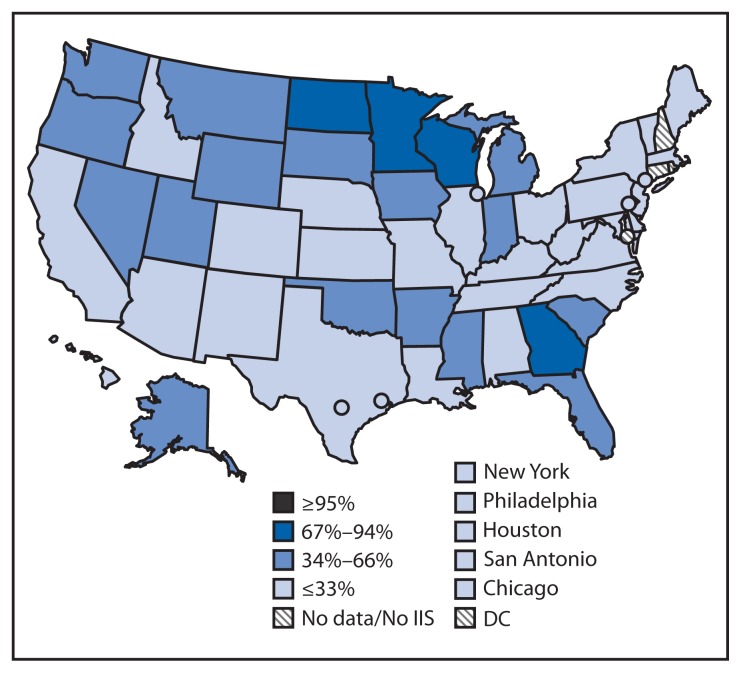
Percentage of adults aged ≥19 years participating in an immunization information system (IIS)^*^ — United States, five cities,^†^ and the District of Columbia (DC), 2012 ^*^ Adult participation is defined as having one or more vaccinations administered to adults aged ≥19 years documented in an IIS. National adult participation = 25%. ^†^Chicago, Illinois; New York, New York; Philadelphia, Pennsylvania; Houston, Texas; and San Antonio, Texas.

Nationally, 19.5 million U.S. children aged <6 years (86.2%) participated in an IIS in 2012. This child participation measure is used to track a *Healthy People 2020* objective (IID-18) to increase to 95% the proportion of children aged <6 years whose immunization records are in fully operational, population-based IIS (7). Child participation in IIS has increased steadily, from 63% in 2006 to 86% in 2012 (1). Of the 54 grantees with available data in 2012, 26 (48%) reported that ≥95% of children aged <6 years in their geographic area participated in their IIS (Figure 1). Nationally, 57.8 million U.S. adults aged ≥19 years (24.5%) participated in an IIS in 2012 (Figure 2). Two IIS did not collect immunization information for adults. The Connecticut IIS includes only children aged <6 years, and the Rhode Island IIS includes only persons aged <19 years. Adult participation in IIS among the remaining 52 grantees responding in 2012 ranged from 0.5% (Houston) to 85.4% (Minnesota).

## Functional Standards for IIS

**FIGURE 3:**
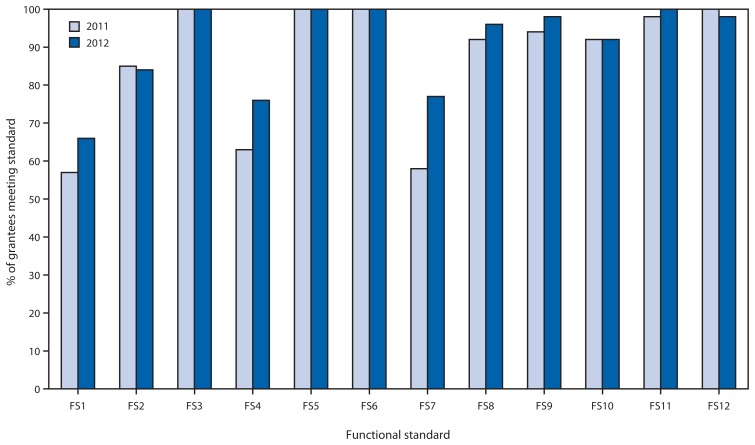
Percentage of immunization program grantees meeting National Vaccine Advisory Committee (NVAC) functional standards (FS) for immunization information systems (IIS),^*^ United States, 2011 and 2012 ^*^The standards include FS1: presence of a field for data collection of all 18 required NVAC core data elements; FS2: establishment of a newborn birth record within an average time of 6 weeks or less; FS3: ability to access patients’ immunization information from the IIS during a patient encounter; FS4: receiving and processing ≥70% of vaccine and other immunization encounter information within 30 days of vaccine administration; FS5: implementation of a written confidentiality policy; FS6: implementation of a written security policy; FS7: meeting basic Health Level 7 messaging functionality; FS8: presence of forecasting algorithm; FS9: ability to run reminder and recall notifications; FS10: ability to produce immunization coverage reports by providers, age groups, and geographic areas upon request; FS11: ability to produce official immunization records; and FS12: presence of a patient-level de-duplication algorithm. Additional information available at http://www.cdc.gov/vaccines/programs/iis/func-stds-2001.html.

Functional standards for IIS were developed in 2001 and revised in 2007. The standards have been approved by NVAC (3,4) for assessing IIS progress in meeting minimum functionalities. Substantial progress has been made in meeting these functional standards since inception, and in 2012, eight of 12 functional standards had been met by ≥90% of grantees (Figure 3). Increases were observed during 2011–2012 in the percentage of grantees meeting three of the four remaining functional standards. The percentage of grantees meeting functional standard (FS) 1 (i.e., reporting the presence of fields in their IIS for 18 required NVAC core data elements) increased from 57% in 2011 to 65% in 2012. Completeness of core data elements has been reported on previously (1). The percentage of grantees meeting FS 4 (i.e., percentage of grantees who reported receiving and processing ≥70% of vaccine and other immunization encounter information within 30 days of vaccine administration) increased from 63% in 2011 to 76% in 2012. The percentage of grantees meeting FS 7 (i.e., meeting basic HL 7 functionality§) increased from 58% in 2011 to 77% in 2012. The percentage of grantees meeting more advanced HL7 functionality¶ increased from 35% in 2011 to 37% in 2012. In 2012, 37% (19) of grantees were sending and receiving any HL7 v.2.5.1 messages, an increase from 17.3% (9) of grantees in 2011. The percentage of grantees meeting FS 2 (i.e., reporting the establishment of a birth record within an average time of ≤6 weeks) decreased from 85% in 2011 to 84% in 2012. This slight decline occurred because three grantees who previously met the functional standard in 2011 reported a decrease in timeliness in 2012 resulting from their acceptance of larger amounts of data, which slowed processing times; however, two grantees achieved the functional standard in 2012 who had not previously.

### Editorial Note

Child participation in IIS increased steadily from 2006 to 2012, reaching 86%; adult participation, however, only reached 25% in 2012. Eight of 12 IIS functional standards were met by ≥90% of grantees in 2012. Increases in grantees meeting minimum functional standards for IIS data quality and interoperability, including the presence of core data element fields, timeliness for vaccination records, and HL7 messaging functionality, also have been demonstrated from 2011 to 2012, although challenges remain for IIS to reach their full potential in these areas, and for improving the timeliness of birth records in IIS.

Historically, the primary focus of IIS and immunization programs has been pediatric populations. This focus was warranted because of the increasing complexity of the routine pediatric immunization schedule, mobility of children among different providers resulting in vaccination record scattering (8) that makes tracking and catch-up immunization challenging, and the role of the IIS in supporting the Vaccines for Children program through ordering and inventory management, report generation, and vaccine accountability. Nevertheless, interest is growing in ensuring that adult populations are included and vaccinations tracked in IIS. Adults are vaccinated by multiple and diverse providers, beyond traditional health-care providers (e.g., pharmacies, retail clinics, and subspecialists), and consolidated adult vaccination records maintained by IIS could play an instrumental role in providing clinical point-of-care support and population-level immunization coverage, particularly in special circumstances such as tracking doses administered during an influenza pandemic.

Currently, 53 of 56 immunization program grantees have IIS with lifespan systems, yet adult participation in IIS remains low. Challenges to increase adult participation in IIS include 1) identifying and enrolling the diverse providers that serve adults, 2) a lack of adult immunization reporting mandates in many grantees’ jurisdictions, and 3) competing priorities for state and local immunization programs. To support increased adult provider participation in IIS, CDC is supporting several new initiatives, including partnering with the Veterans Administration, the Indian Health Service, and federal occupational health clinics; providing supplemental funding to IIS Sentinel Sites to support adult provider enrollment and completeness of adult data in IIS as part of pandemic preparedness; and collaborating with the American Immunization Registry Association to better understand barriers and opportunities for pharmacy reporting to IIS. CDC also has initiated the Clinical Decision Support for Immunization (CDSi) project for the adult vaccine schedule, which will provide a single, authoritative, software-independent foundation for development and maintenance of evaluation and forecast systems (9).** By capturing Advisory Committee on Immunization Practices (ACIP) recommendations for adult vaccination in an unambiguous manner, it will improve the uniform representation of vaccination decision guidelines, and the ability to automate vaccine evaluation and forecasting (9). CDSi for the childhood schedule was completed in October 2012 and has already proven successful in clarifying ACIP recommendations and designing new and existing computer systems.

What is already known on this topic?

In 2011, 84% of U.S. children aged <6 years (19.2 million) participated in immunization information systems (IIS).

What is added by this report?

In 2012, 86% of U.S. children aged <6 years participated in IIS. Adult participation (25%) in IIS lags behind. Eight of 12 minimum functional standards for IIS published by the National Vaccine Advisory Committee have been met by ≥90% grantees, but gaps still exist in meeting Health Level 7 (HL7) interoperability and some data quality standards.

What are the implications for public health practice?

To realize the full benefits of IIS, progress is needed to reach lifespan participation in IIS, advanced bidirectional HL7 messaging between IIS and electronic health records, and improved data quality in IIS. Initiatives designed to increase adult participation in IIS, and promote HL7 messaging and electronic health records use among providers, are expected to support progress in these areas.

In addition to capturing the complete population of children and adults within each IIS jurisdiction, IIS must maintain and enhance system functionality to ensure that data quality is high, protect the confidentiality of data, and serve multiple stakeholders. Although IIS have made great strides in implementing functional standards, progress can still be made in areas such as timeliness of record submission, completeness of core data elements, and HL7 functionality. Several ongoing and new initiatives are expected to support these functional standards, including the use of IIS to support vaccine ordering and inventory management, the use of two-dimensional barcodes to record vaccination information, and interoperability between IIS and electronic health records (1). Implementation of stage 2 meaningful use criteria for the Medicare and Medicaid electronic health record incentive program (10), emphasizing use of HL7 version 2.5.1 and promotion of successful, ongoing submission from providers to IIS, is expected to increase child and adult participation in IIS and improve data quality in IIS, including completeness and timeliness of records. Stage 2 implementation was scheduled to launch in October 2013 for hospitals and January 2014 for providers.

The findings in this report are subject to at least two limitations. First, although CDC provides guidance to grantees to validate IISAR responses, data are self-reported and self-validated, which might result in overestimation or underestimation of participation rates. Second, because two of the 56 grantees did not report data during the period studied, the percentage of grantees meeting each of the functional standards might be higher or lower than calculated.

New functional standards for IIS for 2013–2017 have been developed by CDC through a consensus process involving input from IIS managers and technical experts nationwide (5). Those standards are intended to lay a framework for the development of IIS through 2017, and supersede the minimum functional standards for registries adopted by NVAC in 2001. These new functional standards encompass areas within the old functional standards where progress is still being achieved, including timeliness of records submission, completion of core data elements, and HL7 interoperability standards. They also include new areas, such as supporting the Vaccines for Children program and state vaccine purchase programs through vaccine inventory functions and capture of program eligibility at the dose-level, and enhanced data quality through patient- and vaccine-level de-duplication. Grantees meeting and exceeding these new functional standards will lead the way in realizing and demonstrating the full potential of IIS.

## Footnotes

* Chicago, Illinois; New York, New York; Philadelphia, Pennsylvania; Houston, Texas; and San Antonio, Texas.

† Additional information available at http://www.cdc.gov/vaccines/programs/iis/annual-report-iisar/index.html.

§ HL7 is the industry standard to exchange patient information electronically between IIS and electronic medical record systems. Basic HL7 functionality is defined as receiving VXU and sending ACK message types for either HL7 v.2.3.1 or v.2.5.1.

¶ Advanced HL7 functionality is defined as the percentage of grantees who received VXQ and sent VXR and QCK message types for v.2.3.1 and received QBP and sent RSP message types for v.2.5.1.

** Evaluation describes the process of determining if a vaccination has been given at the right time, in the right amount, and with the right product. Forecasting involves recommending when the next dose of a vaccine should be given, based on a patient’s history and the guidelines described in the immunization schedule.
